# Induction of Regulatory T Cells by Intravenous Immunoglobulin: A Bridge between Adaptive and Innate Immunity

**DOI:** 10.3389/fimmu.2015.00469

**Published:** 2015-09-11

**Authors:** Gabriel N. Kaufman, Amir H. Massoud, Marieme Dembele, Madelaine Yona, Ciriaco A. Piccirillo, Bruce D. Mazer

**Affiliations:** ^1^Translational Research in Respiratory Diseases Program, The Research Institute of the McGill University Health Centre, Montreal, QC, Canada; ^2^Laboratory of Cellular and Molecular Immunology, University of Montreal Hospital Research Centre, Montreal, QC, Canada; ^3^Infectious Diseases and Immunity in Global Health Program, The Research Institute of the McGill University Health Centre, Montreal, QC, Canada; ^4^Department of Pediatrics, Faculty of Medicine, McGill University, Montreal, QC, Canada

**Keywords:** intravenous immunoglobulin, regulatory T cell, dendritic cell, autoimmunity, immune modulation, cytokine

## Abstract

Intravenous immunoglobulin (IVIg) is a polyclonal immunoglobulin G preparation with potent immunomodulatory properties. The mode of action of IVIg has been investigated in multiple disease states, with various mechanisms described to account for its benefits. Recent data indicate that IVIg increases both the number and the suppressive capacity of regulatory T cells, a subpopulation of T cells that are essential for immune homeostasis. IVIg alters dendritic cell function, cytokine and chemokine networks, and T lymphocytes, leading to development of regulatory T cells. The ability of IVIg to influence Treg induction has been shown both in animal models and in human diseases. In this review, we discuss data on the potential mechanisms contributing to the interaction between IVIg and the regulatory T-cell compartment.

## Introduction

### Intravenous immunoglobulin

Intravenous immunoglobulin (IVIg) is prepared from polyclonal immunoglobulin G (IgG) purified from pooled plasma samples of several thousand healthy donors. IgG has been the standard treatment for primary immunodeficiency diseases since Bruton’s identification of a patient with agammaglobulinemia in the early 1950s (1). However, Imbach et al. (2), in the early 1980s, demonstrated that administration of high doses of human polyclonal IgG in children with immune deficiency who concomitantly suffered from immune thrombocytopenic purpura (ITP) had a dramatic increase in platelet counts. Since then, there has been a progressive increase in the use of IVIg in patients with a wide variety of autoimmune and inflammatory disorders. IVIg is used as a primary treatment for ITP, Kawasaki Syndrome (KS), Guillain–Barré syndrome, myasthenia gravis, chronic inflammatory demyelinating polyneuropathy, systemic lupus erythematosus, and other autoimmune and neurologic disorders (3). IVIg is commonly used in the prevention or treatment of neonatal sepsis. Rationally, those infants who are premature or suffer from very low birth weights should benefit from the immune supplementation provided by IVIg. However, large randomized clinical trials have failed to show consistent benefit in terms of prevention or outcomes from septic episodes (4–6). Novel preparations enriched in IgM may have some promise, but to date results are inconsistent (7, 8).

Intravenous immunoglobulin is now the most commonly prescribed plasma-based product worldwide (3, 9). This increased use raises questions regarding the long-term viability of this therapy. Considering the high cost and limited availability of this resource, it is imperative to investigate the underlying mechanisms of IVIg in order to tailor the anti-inflammatory response obtained by treatment, allowing for better therapies for inflammatory and autoimmune diseases. Furthermore, this can lead to the development of non-plasma-derived drugs with similar therapeutic benefits.

Many mechanisms explaining the immune-regulatory actions of IVIg have been postulated, including modulation of inhibitory Fc-gamma receptor (FcγR) expression, blockade of activating FcγR on antigen-presenting cells (APCs), interference with cytokine production, inhibition of cell activation, or induction of apoptosis in a variety of immune cells, including dendritic cells (DCs), macrophages, natural killer (NK) cells, and T and B lymphocytes (3, 9, 10). However, a key factor in immune modulation is the ability to counter inflammatory responses with regulatory cells. In this review, we will explore the links between IVIg and regulatory T-cell responses.

### Regulatory T cells

Regulatory T cells (Treg) were initially described in the 1990s as a specialized subpopulation of T cells that maintain immune system homeostasis and tolerance to self-antigens (11, 12). The transcription factor forkhead box P3 (FOXP3) is considered the marker of choice for this cell (13). FOXP3 is a master-switch transcription factor: its expression modifies T cells toward a regulatory phenotype, enabling many of the anti-inflammatory functions of Treg (14). The fundamental property that defines Treg is their ability to transfer immune suppression *in vivo* from one animal to another or *in vitro* from one cell culture to another (15). Based on their developmental or functional differences, Treg are categorized into two main populations: naturally occurring Treg that are generated in the thymus (tTreg) and peripherally induced Treg (pTreg) generated in peripheral lymphoid tissues from non-Treg precursor CD4^+^ cells. While Treg are CD4^+^ T-effector cells with characteristic FOXP3 expression, this is not sufficient to define a cell population as Treg: single-cell flow cytometric sorting experiments have shown the importance of elevated expression of the high-affinity IL-2 receptor, CD25, as a hallmark of Treg (16). Other markers, including HLA-DR, GARP, and low CD127 expression, along with CTLA-4 and Helios, are not entirely consistent or reliable and depend on the activation state of the cell (17). Recent work by Bin Dhuban et al. (18) has identified two cell-surface Treg markers: TIGIT, a novel CD28-related protein, and FCRL3, an Fc-receptor-like glycoprotein, which allow for high-consistency detection of Treg in human peripheral blood mononuclear cells (PBMCs).

Pre-clinical studies have shown that freshly isolated or *ex vivo-*expanded Treg can confer immunological tolerance in subjects with autoimmune and inflammatory disorders (19, 20). However, human Treg infusion therapy has been difficult to implement, and relatively few clinical trials have been initiated (21). Therefore, developing new therapeutic approaches with the capability to modulate the immune system through activation and/or expansion of Treg has been the subject of many recent studies. Several therapeutic immunosuppressive compounds, including rapamycin (22) and glucocorticoids (23), have been identified as promoting the expansion or suppressive activity of Treg.

Intravenous immunoglobulin has been proposed as a treatment that can promote development or activation of Treg in autoimmune diseases (24). Herein, we provide an overview examining if IVIg indeed influences induction of Treg in the context of different inflammatory and autoimmune conditions and discuss mechanisms underlying Treg induction by IVIg.

## Evidence for the Action of IVIg in the Promotion of Treg in Human Clinical Trials

An early clue suggesting regulatory effects of IVIg was the observation that T cells, purified from IVIg-treated individuals, had significant suppressive effects when cultured with proliferating T and B cells (25). Subsequent studies demonstrated that IVIg therapy was associated with enhanced mitogen-induced “suppressor T-cell function” in rheumatoid arthritis (26), ITP (27), and pediatric acquired immune deficiency syndrome (28). More recently, Kessel et al. (29) demonstrated that *in vitro* culture of IVIg with peripheral T cells led to increases in intracellular TGF-β, IL-10, and FOXP3 expression as well as improvement in their suppressive functions when cocultured with effector T cells.

T cells from patients treated with IVIg have been examined for increases in Treg. In Guillain–Barré syndrome, IVIg therapy increases the expression of *FOXP3* and the production of inhibitory cytokines in Treg (30). In systemic lupus erythematosus, IVIg-treated patients show significant increases in Treg numbers; moreover, IVIg appeared to convert naive FOXP3^−^CD25^−^ into activated FOXP3^+^CD25^+^ Treg (31). Consistently, IVIg therapy of EGPA patients increased FOXP3^+^ Treg numbers and production of IL-10 in CD4^+^ T cells (32). In mononeuritis multiplex, a peripheral neuropathy, steroid unresponsive patients treated with IVIg exhibit enhanced populations of Treg (33).

### Mechanisms of action of IVIg in KS

Kawasaki syndrome is an acute systemic vascular inflammation, primarily affecting children. A single IVIg treatment is generally successful in reducing fever and associated disease manifestations (34). Extensive work has focused on characterizing the IVIg-induced Treg response in KS. Burns et al. (35) investigated the link between TNF-α and IVIg therapy in KS, hypothesizing that TNF-α inhibition may decrease cell activation. They determined that infliximab treatment does not interfere with Treg induction by IVIg, finding that the expansion of CD14^+^ CD86^+^ tolerogenic DC correlated with increased Treg after IVIg treatment. They postulate that the IVIg-induced Treg pool secretes IL-10 and responds to the Ig heavy-chain Fc region.

In a subsequent study from the same group, Franco et al. (36) investigated the specificity of IVIg-induced Treg in subacute KS patients. IVIg treatment induced a subset of Treg that expressed high levels of CTLA-4, and secreted IL-10, but not TGF-β. This Treg expansion appeared to be key to controlling vascular inflammation in KS. Cloned Treg expanded *ex vivo* only responded to soluble IgG Fc and not to F(ab)′^2^ fragments, indicating that these Treg were Fc-specific and that the mechanism was likely T-cell receptor (TCR)-dependent. Coculture experiments revealed that the Fc region of IgG was presented in a major histocompatibility complex (MHC)-restricted, TCR-mediated manner by EBV-transformed B cells. Further investigation of the Fc peptide specificities of the tTreg population revealed similar profiles in both IVIg-treated KS patients and in healthy controls, suggesting that Treg responses are functionally inadequate in KS and that this can be reversed by IVIg (37).

In KS patients, IVIg treatment enhances the expression of genes related to Treg activation, including *FOXP3*, *CTLA4*, *GITR*, and *TGFB1*. The expression levels of these genes were significantly lower in KS patients prior to treatment than in healthy controls (38, 39). Ni et al. (40) examined the mechanisms of Treg dysfunction in KS, focusing on microRNAs (miR). While acute KS patients had lower Treg numbers and decreased Treg marker expression, IVIg treatment increased Treg numbers and *FOXP3*, *CTLA4*, and *GITR* gene expression. Treg from untreated KS have down-regulated miR-155 and miR-21 microRNAs; miR-155 down-regulation leads to increased SOCS1 signaling, decreased STAT-5 signaling, and miR-31 microRNA overexpression. IVIg treatment reversed these effects, restoring the SOCS1/STAT5 balance and decreasing miR-31 expression. FOXP3-dependent miR-155 inhibited SOCS1, and STAT3 suppressed miR-21, which down-regulated FOXP3. IVIg treatment of KS patients lowered elevated IL-6 and pSTAT3, restoring miR-21 levels, providing an explanation for the increase in Treg numbers following IVIg infusion.

## Modulatory Effects of IVIg in Animal Models of Inflammatory Disorders via Treg Expansion and Induction

### Role of IVIg in experimental autoimmune encephalomyelitis

In experimental autoimmune encephalomyelitis (EAE), an antigen-driven murine model of multiple sclerosis, IVIg treatment reduced the disease severity scores, promoted the expansion of Treg and enhanced their suppressive capacity, both *in vivo* and *in vitro* (39). Importantly, administration of IVIg failed to confer protection in EAE mice that were depleted of Treg prior to treatment, suggesting a critical role of endogenous Treg in conferring protection by IVIg. In line with these findings, Okuda et al. (41) replicated the effects of IVIg in EAE and showed that sulfonated IVIg was effective in increasing the frequency of Treg.

A potential target for IVIg in EAE is NK cells. NK cells have a wide variety of immunomodulatory functions, interacting with B cells, DC, and Treg (42, 43). Chong et al. (44) hypothesized that in IVIg-treated subjects, NK cells suppress disease by regulating inflammatory T-cell responses. Using an EAE model, they demonstrated that IVIg treatment blocks EAE development and reduces demyelination by diminishing IL-17 and IFN-γ. NK cell depletion by anti-asialo GM1 antibody resulted in the loss of IVIg-mediated protection, and adoptive transfer of IVIg-treated NK cells was as equally protective as IVIg treatment. IVIg-treated NK cells induced CD4^+^ Foxp3^+^ Treg in spleen and draining lymph nodes, which were suppressive to antigen-specific effector T cells in *ex vivo* proliferation assays. Upon further investigation using an *in vitro* coculture system, Treg induction was determined to depend on IL-2 and TGF-β1 production by NK cells. Chong et al. posit that IVIg may promote redistribution of NK cells in peripheral tissues, depending on the inflammatory stimulus. Since NK cells modulate their chemokine receptor expression to facilitate migration to local and peripheral sites of inflammation (45), IVIg may increase NK cell homing to inflammatory microenvironments and secondary lymphoid organs, where they can induce Treg. NK cell costimulatory molecule expression may also drive Treg induction: IL-2 and plate-bound anti-CD16 treatment up-regulate CD86 and OX-40 ligand on NK cells *in vitro* (46). CD86 has been implicated in Treg generation (47), and OX-40 ligand can deliver a survival signal to Treg (48).

### Treg induction in allergen-driven and autoimmune models

We have recently demonstrated, using an ovalbumin-driven murine model of allergic airway disease, that therapeutic administration of IVIg attenuated airway hyper-reactivity (AHR) and alleviated airway inflammation. This was accompanied by induction of highly suppressive, antigen-specific Treg derived from pre-existing T-effector cells. Treg induction was dependent on the interaction of IVIg with CD11c^+^ DC (49). Similarly, in a murine model of ITP, IVIg increased thymic and splenic Treg, accompanied by restoration of platelet counts (50).

Different dosing regimens have been used for IVIg to increase Treg (51). Our laboratory, as well as most other groups, uses high-dose IVIg (2 g/kg), which is analogous to the immunomodulatory dose used in clinical practice (49). Other studies have used typical antibody-replacement doses of 400–800 mg/kg (52). Ramakrishna et al. (53) reported an anti-inflammatory effect using extremely low-dose IVIg (187.5 mg/kg) in a HSV-mediated encephalitis murine model, which was felt to be dependent on enhancement of Treg. This dose range is rarely used clinically, making this work difficult to apply to standard practice. In addition, work from our laboratory and others (38, 54) suggests that a minor fraction of IVIg is required for some, but not all immunomodulatory effects. This will be discussed in more detail below.

## Mechanisms of Action of IVIg in Induction of Treg

The mechanisms by which IVIg induces Treg may involve direct interaction of IgG with T cells, or modulation of other cellular or molecular targets, particularly APCs such as DC and macrophages. IVIg can also interact with other cells, such as B cells or NK cells. In addition, IVIg can modulate the production of proinflammatory cytokines, which may play a role in maintaining T-cell tolerance.

### The effect of IVIg on DC activation

Induction of protective T-cell responses requires naive T cells to receive signals via the TCR, costimulatory molecules, and cytokine receptors. These signals, via cell–cell contact and through soluble mediators, are provided by professional APCs, such as DC. While DC represent the most efficient APC in capturing, processing, and presenting antigens to T cells (55), DC also play an active role in maintaining immune tolerance, as constitutive DC ablation results in spontaneous fatal autoimmunity (56). Tolerogenic DCs are characterized by decreased expression of costimulatory molecules (CD40, CD80, and CD86), decreased antigen presentation (due to reduced MHC class II expression), enhanced expression of coinhibitory molecules (e.g., PD-L1, CTLA-4, and OX-40), and enhanced inhibitory cytokine production (57, 58). This DC subset is essential for maintaining tolerance via extrathymic induction of pTreg and maintenance of pre-existing tTreg (59–62).

Induction of tolerance is critically dependent on the maturation state of DC. An immature DC phenotype is associated with induction, expansion, or enhancement of the suppressive capacity of Treg (63). Direct cell-to-cell interaction of DC and T cells via TCR (64), induction of indolamine-2,3-dioxygenase (IDO) (65), as well as secretion of IL-10, TGF-β, and retinoic acid by DC (66) are all implicated in the peripheral induction or expansion of Treg by DC.

Although both myeloid and plasmacytoid DC may be involved in maintaining peripheral tolerance (67), polyclonal human IgG appears to target CD11c^+^ DC rather than CD11c^−^ plasmacytoid DC (49, 68). We have demonstrated that CD11c^+^ DC from IVIg-treated mice are necessary and sufficient for peripheral induction of Treg in lung and draining thoracic lymph nodes (49). IVIg decreases CD80 and CD86 both *in vitro* and *in vivo*; in addition, adoptively transferred IVIg-treated DC can increase Treg in lungs of antigen-exposed and challenged mice (49, 69).

Intravenous immunoglobulin-exposed CD11c^+^ DCs are less competent in driving lymphocyte proliferation, potentially due to decreased MHC-II and CD80/CD86 expression (68, 70–72). Work from the group of Bazin suggests that internalized IVIg interferes with antigen presentation by competing with antigen peptides for loading on MHC-II molecules in the intracellular MHC-II compartment (MIIC) (73, 74). Inhibition of T-cell responses by reducing antigen presentation may also interfere with the activation of autoreactive pathogenic T cells. In addition, IVIg alters the pattern of DC cytokine production, including up-regulation of inhibitory cytokines, such as IL-10, and down-regulation of proinflammatory cytokines, such as IL-12 and IFN-γ (53, 71, 75).

Proinflammatory cytokines counteract Treg differentiation or decrease Treg suppressive effects. For example, IL-6 secretion from DC is known to abrogate Treg anergy, reverse Treg suppression, and skew Treg differentiation toward Th-17 (76, 77). In contrast, IVIg reduces the production of IL-6 and TNF-α by peripheral blood monocytes (78, 79); it can therefore maintain Treg homeostasis. It is conceivable that IVIg-generated Treg may attenuate DC maturation by anti-inflammatory cytokine production, expanding the inhibitory effects of IVIg by further tolerizing DC in a negative feedback loop.

How IVIg targets DC is still incompletely elucidated, and different mechanisms have been postulated. The effect of polyclonal IgG on DC appears to involve activating FcγR, by triggering immunoreceptor tyrosine-based activation motifs (ITAM) (80). However, both Fc and F(ab′)^2^ fragments of IgG have been shown to suppress DC maturation and modulate DC cytokine production (71). F(ab′)^2^ fragments have been shown to inhibit LPS-induced phosphorylation of extracellular signal-regulated kinase (ERK1/2), an intracellular signaling molecule that mediates the inflammatory response induced by Toll-like receptor (TLR) ligation in DC (81).

Although a full discussion of IVIg-Fcγ receptor biology is beyond the scope of this review, it is important to note that inhibitory FcγRIIB were required for the anti-inflammatory effects of IVIg in murine models of ITP (72), nephrotoxic nephritis (82), and epidermolysis bullosa acquisita (EBA) (54). Similarly, we have found that FcγRIIB is required for IVIg-mediated abrogation of allergic airways disease (Kaufman et al., in preparation). Up-regulation of FcγRIIB expression on DC, and on APC in general, likely plays a role in the suppression of DC activation, although no direct physical interaction between IVIg with this receptor has been reported (83).

De Groot et al. (84) proposed another DC-dependent mechanism by which IVIg promotes Treg expansion. They described promiscuous IgG-derived T-cell epitope peptides (Tregitopes) containing epitopes from both Fc and Fab fragments of the IgG molecule, with the capability of activating Foxp3^+^ Treg. They postulated that these Tregitopes are presented in the context of MHC-II by APC to Treg and contribute to Treg activation and expansion (85, 86). This is consistent with the results of Franco et al. (36) discussed earlier where B cells presented Fc regions of IgG in a MHC-restricted and TCR-mediated manner.

### The anti-inflammatory effects of sialylated IgG and its relationship to Treg development

Human IgG therapy has two consistently used dosing regimens. Patients requiring immune supplementation for immune deficiency typically receive between 400 and 800 mg/kg monthly. After many years of using lower doses, these were deemed ineffective in crossover studies. Individuals requiring immune modulation frequently receive infusion of IVIg containing two to five times the immune supplementation dose. It has therefore been hypothesized that minor fractions of IVIg provide molecules that supply the anti-inflammatory components needed for immune modulation. This has been demonstrated regarding specific neutralizing antibodies, anti-idiotypic antibodies, or anti-apoptosis antibodies.

The Ravetch group developed the concept that the anti-inflammatory properties of IVIg were isolated to an IgG subset characterized by terminal α-2,6-sialylation of the Fc glycan. Specifically, the Fc portion of the IgG molecule contained an N-linked glycan moiety covalently bound to a highly conserved glycosylation site at Asn297 (87, 88). In various clinical scenarios, lower serum levels of sialylated IgG were found in individuals with systemic lupus erythematosus or juvenile-onset rheumatoid arthritis when compared with healthy controls (89–91). This sialylated fraction of IVIg (saIVIg) makes up roughly 1–2% of the total IgG in pooled therapeutic preparations. In proof-of-concept studies, saIVIg was therapeutically effective in animal models of rheumatoid arthritis (92), ITP (93), and allergic airways disease (94) at doses 10 times lower than unfractionated IVIg.

The mechanism of action of saIVIg is still under investigation. Kaneko et al. (87) proposed that saIVIg interacts with DC-SIGN (DC-specific intercellular adhesion molecule-3 grabbing non-integrin) on human DC or the murine ortholog SIGN-R1 (specific intracellular adhesion molecule-3 grabbing non-integrin homolog-related 1) on murine splenic macrophages. This triggers increased expression of the inhibitory Fc receptor FcγRIIB (88). This may contribute to the induction and expansion of Treg; FcγRIIB-deficient mice are incapable of generating Treg in a model of mucosal antigen tolerance (95). Guilliams et al. reviewed the role of FcγRIIB in IVIg therapy and suggested that IVIg increases FcγRIIB expression in inflamed tissues during the effector phase of the immune response (96).

Using experimental models of multiple sclerosis (EAE) and serum-induced arthritis, Fiebiger et al. (97) recently reported that saIVIg Fc confers protective effects in T-cell-mediated and antibody-mediated diseases. They developed a mutated IgG Fc construct (F241A), which had a similar structure to saIVIg Fc, but displayed DC-SIGN binding independent of sialylation. Both saIVIg Fc and F241A IgG Fc alleviated arthritis and EAE by inducing Treg expansion and activation, up-regulating FcγRIIB on effector macrophages, and suppressing Th17 and Th1 responses. The anti-inflammatory responses required expression of DC-SIGN as well as secretion of IL-33 by macrophages.

Washburn et al. described a novel hypersialylated IgG derivative, tetra-Fc-sialylated IVIg (s4-IVIg), which was maximally sialylated but lacked advanced glycation end products (AGEs) that are hazardous to human health. s4-IVIg was efficacious in animal models of arthritis, ITP, and EBA (91). These results substantiate data obtained by Schwab et al. (54) who demonstrated requirements for IgG sialylation and FcγRIIB expression in their disease models.

As IVIg is a heterogeneous compound, it is not surprising that non-sialylated IgG is also biologically active. Othy et al. demonstrated the effects of IVIg on Th17 and Treg cells independent of Fc sialylation (98). Similarly, there are studies using different murine models of ITP (99, 100) and rheumatoid arthritis (101), which did not require sialylated IgG. Differences in strains or induction of pathological conditions in various murine models are reasons for the discrepancies in the dependence on sialylation. To obtain more definitive results, it will be critical to evaluate the role of minor IgG fractions in subjects with inflammatory and autoimmune diseases.

### IVIg binds C-type lectin receptors on DCs

DC-specific intercellular adhesion molecule-3 grabbing non-integrin and SIGN-R1 are C-type lectin receptors, which bind mannosylated and fucosylated structures, such as HIV envelope protein gp120 (102). While ligation of DC-SIGN by mannose-expressing pathogens stimulates proinflammatory cytokine secretion by DC, fucose-expressing pathogens or synthetic fucose-containing ligands inhibit LPS-induced production of IL-6 and IL-12 and stimulate the secretion of anti-inflammatory IL-10 by DC (103). Hence, ligation of these innate receptors by saIVIg may regulate cytokine production by DC and therefore contribute to Treg homeostasis. Smits et al. (104) showed that binding of *Lactobacillus reuteri* and *Lactobacillus casei* bacteria to DC-SIGN on monocyte-derived DC drove the development of Treg. These Treg produced increased levels of IL-10 and were capable of inhibiting the proliferation of bystander T cells in an IL-10-dependent fashion.

Work from the group of Ravetch and other investigators suggests that the conformational changes in IgG molecules induced by sialylation lead to a reduction in binding affinity of IgG to FcγR by masking the FcγR binding site (105). Furthermore, sialylation exposes a binding site on IgG for carbohydrate-binding C-type lectin receptors such as DC-SIGN or SIGN-R1 (89, 97, 106). In contrast, the group of Crispin were unable to reproduce these findings, claiming that sialylation of IgG does not result in conformational changes to the IgG molecule or increases in binding affinity of IgG to DC-SIGN (107, 108). They suggest that cross-linking of sialic-acid-binding Siglecs (sialic acid binding Ig-like lectins), such as CD22, and direct binding of Fc receptors induce inhibitory signaling through immunoreceptor tyrosine-based inhibition motif (ITIM) pathways. We have recently described that IVIg efficiently modifies DCs to induce regulatory T cells in the absence of activating FcγR (94). It is worth noting that the sialylation of IgG is not restricted to the Fc fragment, and a high proportion of sialic acid residues on the F(ab′)^2^ fragments of IgG has been identified recently by Kasemann et al. (109).

In addition to DC-SIGN, other C-type lectin receptors may interact with IVIg and contribute to induction and/or expansion of Treg. We have recently reported (94) that saIVIg specifically interacts with the C-type lectin dendritic cell immunoreceptor (DCIR) on CD11c^+^ DC. This appears to lead to internalization of IgG into DC and is associated with inhibitory signaling in ligated DC that consequently results in the peripheral induction of Foxp3^+^ Treg. The contribution of DCIR^+^ DC in the induction and expansion of Treg has been demonstrated in previous studies, although not in the context of IVIg therapy. Yamazaki et al. (110) showed that two subsets of CD8^+^CD205^+^ and CD8^−^DCIR^+^ DC differentiate peripheral Foxp3^+^ Treg, in part through the endogenous production of TGF-β. These data indicate that multiple C-lectin receptors are implicated in the generation of tolerogenic DC by IVIg.

### Interaction of IVIg with Treg

Direct interaction of polyclonal IgG with Treg may represent another mechanism by which IVIg can induce tolerance. Kessel et al. (29) demonstrated that IVIg increases expression of intracellular FOXP3, TGF-β, and IL-10 when added to culture with human CD4^+^ T cells. IgG was shown to bind to both human and mouse Treg (39, 111), which increased FOXP3 expression, accompanied by augmented *ex vivo* suppressive function. IVIg stimulated phosphorylation of ZAP-70 in Treg (111), which is known to enhance suppressive activity (112).

Additionally, interaction of IgG with effector T cells can affect the balance of cytokine production, mainly by down-regulating proinflammatory cytokines, such as IL-2, IFN-γ, and TNF-α, and increasing inhibitory cytokines (113, 114). Early work (115) on cytokine networks elucidated that IVIg abrogated production of both Th1- and Th2-type proinflammatory cytokines from PBMC in culture. Maddur et al. (116) demonstrated the reciprocal enhancement of Treg differentiation compared to inhibition of Th17 differentiation in culture, in association with decreases in Th17 effector cytokines (IL-17A, IL-17F, IL-21, and CCL20). In clinical trials, two groups have investigated the effect of IVIg therapy on the profile of intracellular cytokine expression in T cells. In ITP patients who were responsive to IVIg therapy, there was increased production of IL-10 and TGF-β by CD4^+^ T cells as well as decreased Th-1 cytokine production (117, 118).

Experiments from our laboratory could not confirm direct action of IVIg on T cells on the induction of Treg. We examined naive CD4^+^ Foxp3^−^ T cells from Foxp3-GFP reporter mice in the absence of APC. Pre-treatment of these cells with IVIg, followed by coculture with DC and a source of antigen, did not result in the induction of Foxp3 expression, whereas IVIg pretreatment of DC prior to coculture induced Treg *ex vivo*. Further, we found that (49), in allergic airways disease, Treg induction required CD11c^+^ DC both *in vitro* and *in vivo*, suggesting that the DC compartment is the main target of IVIg in our system. We therefore hypothesize that IVIg first tolerizes DC, which in turn induce Treg.

Modification of chemokine or chemokine receptors on circulating leukocytes is another potential mode of action of IVIg, which would lead to recruitment of cells to specific tissue sites. Evidence suggests that Treg compartmentalization and trafficking are tissue- or organ-specific and that distinct chemokine receptor and integrin expression may contribute to selective trafficking of Treg to inflammatory microenvironments (119). For instance, expression of chemokine receptors CCR4 and CCR8 are required on Treg for tissue homing (120). Treg may switch their homing receptor expression profiles depending on the direction of their trafficking. A majority of Treg found in secondary lymphoid tissues express CD62L and CCR7 (121). Moreover, while both effector and regulatory T cells might express similar patterns of chemokine receptors, both subsets may compete for interaction with APC or access to the site of inflammation.

We have demonstrated in a mouse model of ovalbumin-driven allergic airway disease that IVIg specifically increases the expression of CCR4 on the induced Treg population, suggesting their enhanced ability to recruit to the site of inflammation. Additionally, we found that expression of CD62L, which acts as a homing receptor for lymphocytes entering secondary lymphoid tissues, is decreased in Treg isolated from inflamed lung tissues (49). In a murine model of ITP, Treg compartmentalization was also modified by IVIg therapy (50), stressing the potential for action of IVIg on chemokine receptor expression. It is unclear if this action is direct or indirect, via signals from APC.

## Conclusion

Intravenous immunoglobulin is an extremely complex preparation that contains a multitude of biologically active moieties: it likely achieves immunomodulation through a number of synergistic mechanisms, which provide positive therapeutic effects. The immune-regulatory effects of IVIg appear to be pleiotropic and involve different stages of the inflammatory cascade, with a complex interplay of IgG molecules with different cells and mediators.

In this review, we describe potential mechanisms behind the actions of IVIg in the generation and differentiation of Treg. Recent findings reinforce the efficacy of IVIg in the enhancement of Treg in various autoimmune disorders. The action of IVIg in the modulation of Treg, and the consequent maintenance of immune tolerance, provides a rationale for therapeutic approaches specifically targeting this axis of the immune system. This also renews interest in developing alternative treatments, such as Tregitopes or monoclonal antibodies, for refractory inflammatory and autoimmune diseases, which are often associated with deficiencies in Treg and are difficult to manage with conventional therapeutic approaches.

**Figure 1:**
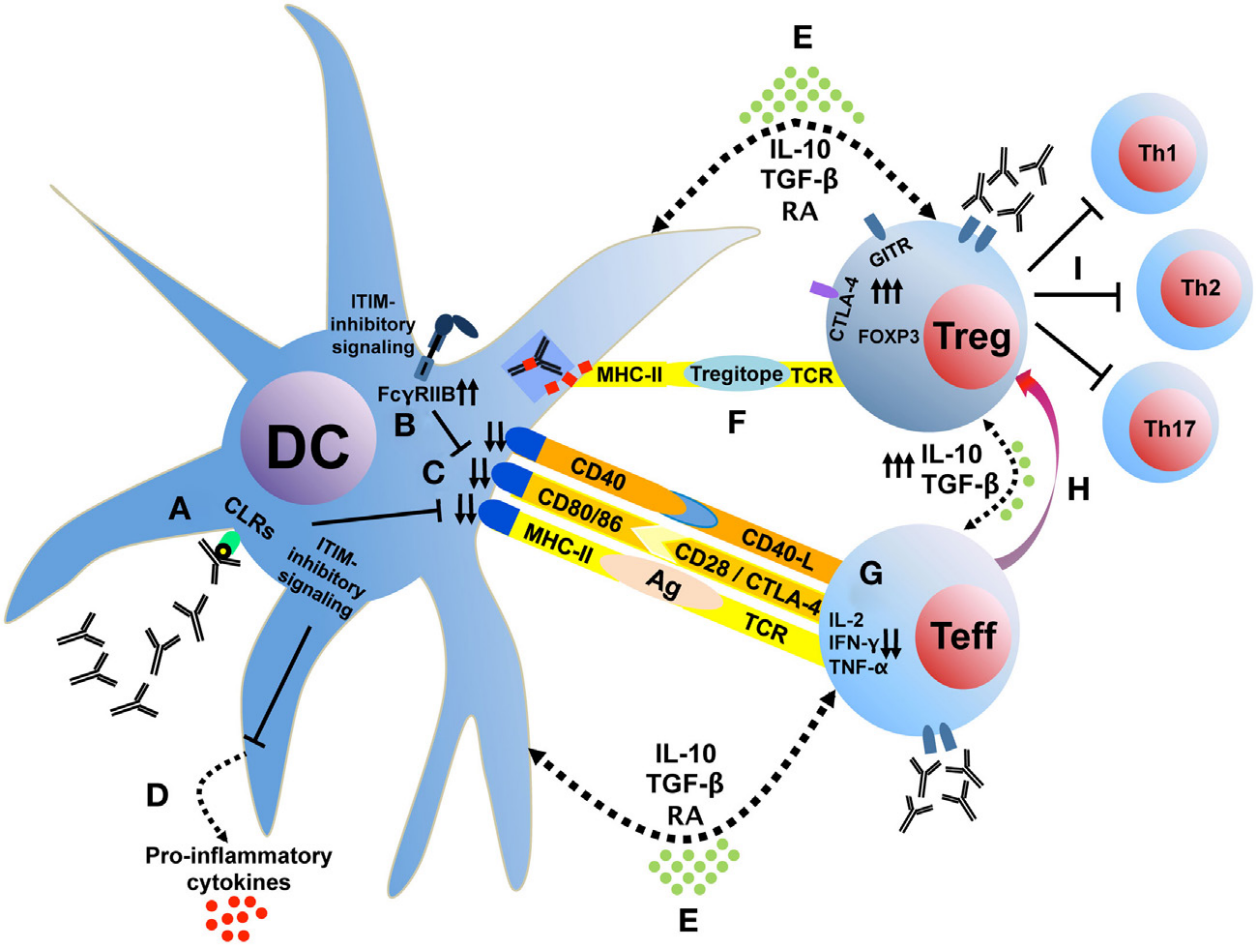
**IVIg tolerizes DC, which interacts with T cells to induce Treg**. Sialylated IVIg ligates C-type lectin receptors on DC **(A)**, which induces FcγRIIB expression **(B)** and reduces costimulatory molecule expression **(C)** and proinflammatory cytokine secretion **(D)**. Anti-inflammatory cytokine and mediator production **(E)** and presentation of IgG regulatory epitopes **(F)** decrease proinflammatory cytokine production in naive effector T cells **(G)** and generate Treg from non-Treg precursors **(H)**. These Treg inhibit effector Th1, Th2, and Th17 cell proliferation and activity **(I)** in the inflammatory microenvironment and secrete anti-inflammatory cytokines **(E)** that tolerize DC. CLRs, C-type lectin receptors; GITR, glucocorticoid-induced TNFR family related gene; ITIM, immunoreceptor tyrosine-based inhibition motif; RA, retinoic acid.

The effects of IVIg on the potentiation of Treg appear to involve the interaction of IgG with APC and potentially T cells and are dependent on the modulation of cytokine networks within different immune cell types. Based on our own studies and the conclusions of this review, we suggest a set of potential cellular mechanisms, which are summarized schematically in Figure 1. Initially, saIVIg binds C-type lectin receptors on DCs (Figure 1A), which induces inhibitory FcγRIIB expression on DC or on effector macrophages (Figure 1B), thus potentiating the activation threshold of the adaptive arm of the immune system. The associated inhibitory receptor signaling renders the DC tolerogenic, reducing DC costimulatory molecule expression (Figure 1C) and proinflammatory cytokine secretion (Figure 1D). Anti-inflammatory cytokine and mediator production by both DC and Treg (Figure 1E) and presentation of IgG regulatory epitopes (Figure 1F) to Treg by DC decrease proinflammatory cytokine production in naive effector T cells (Figure 1G) and generate Treg from non-Treg precursors (Figure 1H). These Treg inhibit effector Th1, Th2, and Th17 cell proliferation and activity (Figure 1I) in the inflammatory microenvironment and secrete anti-inflammatory cytokines (Figure 1E) that tolerize DC. In addition, IVIg-mediated modulation of chemokine or chemokine receptor expression in T-cell subsets might contribute to the homeostasis or regulation of trafficking of Treg, although proper functional characterization is needed. NK cells play a role in processing of innate antigens and have multiple known ITIM-linked receptors: IVIg may target NK cells to directly induce Treg by cytokine production or cell–cell contact (discussed earlier). IVIg-treated NK cells may also induce antibody-dependent cellular cytotoxicity of mature DC, which reduces antigen presentation and inhibits proinflammatory effector T-cell function (122).

Interaction of sialylated IgG with C-type lectin receptors triggers an inhibitory response in ligated cells that might consequently provide the required signals for maintaining immune tolerance. In this review, we bring evidence that shows the association of this interaction with the promotion of Treg. However, more investigation is still required to elucidate the beneficial effects of IVIg in modulation of Treg, particularly in clinical trials. Further avenues of research include identifying specific cellular markers or phenotypic patterns associated with DC tolerogenicity as well as precise characterization of the IVIg-generated Treg population.

## Author Contributions

GK, AM, MD, and MY surveyed the literature and wrote the article. GK, CP, and BM revised the article. All authors approved the final version of this article for publication and accept responsibility for the integrity of the work.

## Conflict of Interest Statement

The authors declare that the research was conducted in the absence of any commercial or financial relationships that could be construed as a potential conflict of interest.

## Abbreviations

APC, antigen-presenting cell; CLRs, C-type lectin receptors; DC, dendritic cell; EAE, experimental autoimmune encephalitis; F(ab), fragment antibody-binding region; Fc, fragment crystallizable region; FcγR, Fc-gamma receptor; GFP, green fluorescent protein; IgG, immunoglobulin G; ITAM, immunoreceptor tyrosine-based activation motif; ITIM, immunoreceptor tyrosine-based inhibition motif; ITP, immune thrombocytic purpura; IVIg, intravenous immunoglobulin; KS, Kawasaki syndrome; MHC, major histocompatibility complex; NK, natural killer; saIVIg, sialylated fraction of IVIg; TCR, T-cell receptor; Treg, regulatory T cell.

## References

[1] Bruton OC Agammaglobulinemia. Pediatrics (1952) 9(6):722–8. 14929630

[2] Imbach P Barandun S Baumgartner C Hirt A Hofer F Wagner HP. High-dose intravenous gammaglobulin therapy of refractory, in particular idiopathic thrombocytopenia in childhood. Helv Paediatr Acta (1981) 36(1):81–6. 6785258

[3] Nimmerjahn F Ravetch JV. Anti-inflammatory actions of intravenous immunoglobulin. Annu Rev Immunol (2008) 26:513–33. 10.1146/annurev.immunol.26.021607.090232 18370923

[4] Group IC Brocklehurst P Farrell B King A Juszczak E Darlow B Treatment of neonatal sepsis with intravenous immune globulin. N Engl J Med (2011) 365(13):1201–11. 10.1056/NEJMoa1100441 21962214

[5] Ohlsson A Lacy JB. Intravenous immunoglobulin for preventing infection in preterm and/or low birth weight infants. Cochrane Database Syst Rev (2013) 7:CD000361. 10.1002/14651858.CD000361.pub3 23821390

[6] Ohlsson A Lacy JB. Intravenous immunoglobulin for suspected or proven infection in neonates. Cochrane Database Syst Rev (2013) 7:CD001239. 10.1002/14651858.CD001239.pub4 23821359

[7] Akdag A Dilmen U Haque K Dilli D Erdeve O Goekmen T. Role of pentoxifylline and/or IgM-enriched intravenous immunoglobulin in the management of neonatal sepsis. Am J Perinatol (2014) 31(10):905–12. 10.1055/s-0033-1363771 24515621

[8] Capasso L Borrelli AC Parrella C Lama S Ferrara T Coppola C Are IgM-enriched immunoglobulins an effective adjuvant in septic VLBW infants? Ital J Pediatr (2013) 39:63. 10.1186/1824-7288-39-63 24098953 PMC3851812

[9] Kazatchkine MD Kaveri SV Immunomodulation of autoimmune and inflammatory diseases with intravenous immune globulin. N Engl J Med (2001) 345(10):747–55. 10.1056/NEJMra993360 11547745

[10] Gold R Stangel M Dalakas MC. Drug Insight: the use of intravenous immunoglobulin in neurology – therapeutic considerations and practical issues. Nat Clin Pract Neurol (2007) 3(1):36–44. 10.1038/ncpneuro0376 17205073

[11] Sakaguchi S Sakaguchi N Asano M Itoh M Toda M Immunologic self-tolerance maintained by activated T cells expressing IL-2 receptor alpha-chains (CD25). Breakdown of a single mechanism of self-tolerance causes various autoimmune diseases. J Immunol (1995) 155(3):1151–64. 7636184

[12] Tang Q Bluestone JA. The Foxp3+ regulatory T cell: a jack of all trades, master of regulation. Nat Immunol (2008) 9(3):239–44. 10.1038/ni1572 18285775 PMC3075612

[13] Piccirillo CA Shevach EM. Naturally-occurring CD4+CD25+ immunoregulatory T cells: central players in the arena of peripheral tolerance. Semin Immunol (2004) 16(2):81–8. 10.1016/j.smim.2003.12.003 15036231

[14] Hori S Nomura T Sakaguchi S. Control of regulatory T cell development by the transcription factor Foxp3. Science (2003) 299(5609):1057–61. 10.1126/science.1079490 12522256

[15] Vigouroux S Yvon E Biagi E Brenner MK. Antigen-induced regulatory T cells. Blood (2004) 104(1):26–33. 10.1182/blood-2004-01-0182 15026316

[16] d’Hennezel E Yurchenko E Sgouroudis E Hay V Piccirillo CA. Single-cell analysis of the human T regulatory population uncovers functional heterogeneity and instability within FOXP3+ cells. J Immunol (2011) 186(12):6788–97. 10.4049/jimmunol.1100269 21576508

[17] Bin Dhuban K Kornete M Mason SE Piccirillo CA. Functional dynamics of Foxp3(+) regulatory T cells in mice and humans. Immunol Rev (2014) 259(1):140–58. 10.1111/imr.12168 24712464

[18] Bin Dhuban K d’Hennezel E Nashi E Bar-Or A Rieder S Shevach EM Coexpression of TIGIT and FCRL3 identifies Helios+ human memory regulatory T cells. J Immunol (2015) 194(8):3687–96. 10.4049/jimmunol.1401803 25762785 PMC4610024

[19] Pilat N Baranyi U Klaus C Jaeckel E Mpofu N Wrba F Treg-therapy allows mixed chimerism and transplantation tolerance without cytoreductive conditioning. Am J Transplant (2010) 10(4):751–62. 10.1111/j.1600-6143.2010.03018.x 20148810 PMC2856406

[20] Xu W Lan Q Chen M Chen H Zhu N Zhou X Adoptive transfer of induced-Treg cells effectively attenuates murine airway allergic inflammation. PLoS One (2012) 7(7):e40314. 10.1371/journal.pone.0040314 22792275 PMC3392250

[21] Riley JL June CH Blazar BR. Human T regulatory cell therapy: take a billion or so and call me in the morning. Immunity (2009) 30(5):656–65. 10.1016/j.immuni.2009.04.006 19464988 PMC2742482

[22] Battaglia M Stabilini A Roncarolo MG. Rapamycin selectively expands CD4+CD25+FoxP3+ regulatory T cells. Blood (2005) 105(12):4743–8. 10.1182/blood-2004-10-3932 15746082

[23] Ray A Khare A Krishnamoorthy N Qi Z Ray P. Regulatory T cells in many flavors control asthma. Mucosal Immunol (2010) 3(3):216–29. 10.1038/mi.2010.4 20164832 PMC3039023

[24] Bayry J Mouthon L Kaveri SV Intravenous immunoglobulin expands regulatory T cells in autoimmune rheumatic disease. J Rheumatol (2012) 39(2):450–1. 10.3899/jrheum.111123 22298280 PMC3623090

[25] Durandy A Fischer A Griscelli C. Dysfunctions of pokeweed mitogen-stimulated T and B lymphocyte responses induced by gammaglobulin therapy. J Clin Invest (1981) 67(3):867–77. 10.1172/JCI110104 6162859 PMC370638

[26] Sany J Clot J Bonneau M Andary M. Immunomodulating effect of human placenta-eluted gamma globulins in rheumatoid arthritis. Arthritis Rheum (1982) 25(1):17–24. 10.1002/art.1780250103 7066033

[27] Delfraissy JF Tchernia G Laurian Y Wallon C Galanaud P Dormont J. Suppressor cell function after intravenous gammaglobulin treatment in adult chronic idiopathic thrombocytopenic purpura. Br J Haematol (1985) 60(2):315–22. 10.1111/j.1365-2141.1985.tb07417.x 2408658

[28] Gupta A Novick BE Rubinstein A. Restoration of suppressor T-cell functions in children with AIDS following intravenous gamma globulin treatment. Am J Dis Child (1986) 140(2):143–6. 2936236 10.1001/archpedi.1986.02140160061033

[29] Kessel A Ammuri H Peri R Pavlotzky ER Blank M Shoenfeld Y Intravenous immunoglobulin therapy affects T regulatory cells by increasing their suppressive function. J Immunol (2007) 179(8):5571–5. 10.4049/jimmunol.179.8.5571 17911644

[30] Chi LJ Wang HB Zhang Y Wang WZ. Abnormality of circulating CD4(+)CD25(+) regulatory T cell in patients with Guillain-Barre syndrome. J Neuroimmunol (2007) 192(1–2):206–14. 10.1016/j.jneuroim.2007.09.034 17997492

[31] Barreto M Ferreira RC Lourenco L Moraes-Fontes MF Santos E Alves M Low frequency of CD4+CD25+ Treg in SLE patients: a heritable trait associated with CTLA4 and TGFbeta gene variants. BMC Immunol (2009) 10:5. 10.1186/1471-2172-10-5 19173720 PMC2656467

[32] Tsurikisawa N Saito H Oshikata C Tsuburai T Akiyama K. High-dose intravenous immunoglobulin treatment increases regulatory T cells in patients with eosinophilic granulomatosis with polyangiitis. J Rheumatol (2012) 39(5):1019–25. 10.3899/jrheum.110981 22467925

[33] Tsurikisawa N Taniguchi M Saito H Himeno H Ishibashi A Suzuki S Treatment of Churg-Strauss syndrome with high-dose intravenous immunoglobulin. Ann Allergy Asthma Immunol (2004) 92(1):80–7. 10.1016/S1081-1206(10)61714-0 14756469

[34] Burns JC Glodé MP Kawasaki syndrome. Lancet (2004) 364(9433):533–44. 10.1016/S0140-6736(04)16814-1 15302199

[35] Burns JC Song Y Bujold M Shimizu C Kanegaye JT Tremoulet AH Immune-monitoring in Kawasaki disease patients treated with infliximab and intravenous immunoglobulin. Clin Exp Immunol (2013) 174(3):337–44. 10.1111/cei.12182 23901839 PMC3826300

[36] Franco A Touma R Song Y Shimizu C Tremoulet AH Kanegaye JT Specificity of regulatory T cells that modulate vascular inflammation. Autoimmunity (2014) 47(2):95–104. 10.3109/08916934.2013.860524 24490882

[37] Burns JC Touma R Song Y Padilla RL Tremoulet AH Sidney J Fine specificities of natural regulatory T cells after IVIG therapy in patients with Kawasaki disease. Autoimmunity (2015) 48(3):181–8. 10.3109/08916934.2015.1027817 25822882 PMC4784966

[38] Anthony RM Ravetch JV. A novel role for the IgG Fc glycan: the anti-inflammatory activity of sialylated IgG Fcs. J Clin Immunol (2010) 30(Suppl 1):S9–14. 10.1007/s10875-010-9405-6 20480216

[39] Ephrem A Chamat S Miquel C Fisson S Mouthon L Caligiuri G Expansion of CD4+CD25+ regulatory T cells by intravenous immunoglobulin: a critical factor in controlling experimental autoimmune encephalomyelitis. Blood (2008) 111(2):715–22. 10.1182/blood-2007-03-079947 17932250

[40] Ni FF Li CR Li Q Xia Y Wang GB Yang J. Regulatory T cell microRNA expression changes in children with acute Kawasaki disease. Clin Exp Immunol (2014) 178(2):384–93. 10.1111/cei.12418 25039241 PMC4233387

[41] Okuda S Kamei S Harano S Shinya N Hayashida K Sasaki T. [Enhancement of regulatory T cell induction by intravenous S-sulfonated Immunoglobulin during the treatment of experimental autoimmune encephalomyelitis]. Yakugaku Zasshi (2012) 132(2):243–9. 10.1248/yakushi.132.243 22293707

[42] Bao Y Han Y Chen Z Xu S Cao X. IFN-alpha-producing PDCA-1+ Siglec-H- B cells mediate innate immune defense by activating NK cells. Eur J Immunol (2011) 41(3):657–68. 10.1002/eji.201040840 21287550

[43] Bergmann C Wild CA Narwan M Lotfi R Lang S Brandau S. Human tumor-induced and naturally occurring Treg cells differentially affect NK cells activated by either IL-2 or target cells. Eur J Immunol (2011) 41(12):3564–73. 10.1002/eji.201141532 21905023

[44] Chong WP Ling MT Liu Y Caspi RR Wong WM Wu W Essential role of NK cells in IgG therapy for experimental autoimmune encephalomyelitis. PLoS One (2013) 8(4):e60862. 10.1371/journal.pone.0060862 23577171 PMC3618232

[45] Moretta A. Natural killer cells and dendritic cells: rendezvous in abused tissues. Nat Rev Immunol (2002) 2(12):957–65. 10.1038/nri956 12461568

[46] Zingoni A Sornasse T Cocks BG Tanaka Y Santoni A Lanier LL. Cross-talk between activated human NK cells and CD4+ T cells via OX40-OX40 ligand interactions. J Immunol (2004) 173(6):3716–24. 10.4049/jimmunol.173.6.3716 15356117

[47] Liang S Alard P Zhao Y Parnell S Clark SL Kosiewicz MM. Conversion of CD4+ CD25- cells into CD4+ CD25+ regulatory T cells in vivo requires B7 costimulation, but not the thymus. J Exp Med (2005) 201(1):127–37. 10.1084/jem.20041201 15630140 PMC2212775

[48] Griseri T Asquith M Thompson C Powrie F. OX40 is required for regulatory T cell-mediated control of colitis. J Exp Med (2010) 207(4):699–709. 10.1084/jem.20091618 20368580 PMC2856021

[49] Massoud AH Guay J Shalaby KH Bjur E Ablona A Chan D Intravenous immunoglobulin attenuates airway inflammation through induction of forkhead box protein 3-positive regulatory T cells. J Allergy Clin Immunol (2012) 129(6):1656–65. 10.1016/j.jaci.2012.02.050 22564681

[50] Aslam R Hu Y Gebremeskel S Segel GB Speck ER Guo L Thymic retention of CD4+CD25+FoxP3+ T regulatory cells is associated with their peripheral deficiency and thrombocytopenia in a murine model of immune thrombocytopenia. Blood (2012) 120(10):2127–32. 10.1182/blood-2012-02-413526 22760780

[51] Kerr J Quinti I Eibl M Chapel H Spaeth PJ Sewell WC Is dosing of therapeutic immunoglobulins optimal? – A review of a 3-decade long debate in Europe. Front Immunol (2014) 5:629. 10.3389/fimmu.2014.00629 25566244 PMC4263903

[52] Roifman CM Levison H Gelfand EW. High-dose versus low-dose intravenous immunoglobulin in hypogammaglobulinaemia and chronic lung disease. Lancet (1987) 1(8541):1075–7. 10.1016/S0140-6736(87)90494-6 2883406

[53] Ramakrishna C Newo AN Shen YW Cantin E. Passively administered pooled human immunoglobulins exert IL-10 dependent anti-inflammatory effects that protect against fatal HSV encephalitis. PLoS Pathog (2011) 7(6):e1002071. 10.1371/journal.ppat.1002071 21655109 PMC3107211

[54] Schwab I Mihai S Seeling M Kasperkiewicz M Ludwig RJ Nimmerjahn F. Broad requirement for terminal sialic acid residues and FcgammaRIIB for the preventive and therapeutic activity of intravenous immunoglobulins in vivo. Eur J Immunol (2014) 44(5):1444–53. 10.1002/eji.201344230 24505033

[55] Cines DB Blanchette VS Immune thrombocytopenic purpura. N Engl J Med (2002) 346(13):995–1008. 10.1056/NEJMra010501 11919310

[56] Ohnmacht C Pullner A King SB Drexler I Meier S Brocker T Constitutive ablation of dendritic cells breaks self-tolerance of CD4 T cells and results in spontaneous fatal autoimmunity. J Exp Med (2009) 206(3):549–59. 10.1084/jem.20082394 19237601 PMC2699126

[57] Adorini L. Tolerogenic dendritic cells induced by vitamin D receptor ligands enhance regulatory T cells inhibiting autoimmune diabetes. Ann N Y Acad Sci (2003) 987:258–61. 10.1111/j.1749-6632.2003.tb06057.x 12727648

[58] Steinman RM Hawiger D Nussenzweig MC. Tolerogenic dendritic cells. Annu Rev Immunol (2003) 21:685–711. 10.1146/annurev.immunol.21.120601.141040 12615891

[59] Kushwah R Hu J Role of dendritic cells in the induction of regulatory T cells. Cell Biosci (2011) 1(1):20 10.1186/2045-3701-1-20 21711933 PMC3125210

[60] Zou T Caton AJ Koretzky GA Kambayashi T. Dendritic cells induce regulatory T cell proliferation through antigen-dependent and -independent interactions. J Immunol (2010) 185(5):2790–9. 10.4049/jimmunol.0903740 20686126

[61] Sun Y Brown NK Ruddy MJ Miller ML Lee Y Wang Y B and T lymphocyte attenuator tempers early infection immunity. J Immunol (2009) 183(3):1946–51. 10.4049/jimmunol.0801866 19587015 PMC2895307

[62] Brandl C Ortler S Herrmann T Cardell S Lutz MB Wiendl H. B7-H1-deficiency enhances the potential of tolerogenic dendritic cells by activating CD1d-restricted type II NKT cells. PLoS One (2010) 5(5):e10800. 10.1371/journal.pone.0010800 20520738 PMC2875405

[63] Kubo T Hatton RD Oliver J Liu X Elson CO Weaver CT. Regulatory T cell suppression and anergy are differentially regulated by proinflammatory cytokines produced by TLR-activated dendritic cells. J Immunol (2004) 173(12):7249–58. 10.4049/jimmunol.173.12.7249 15585847

[64] Bhattacharya P Gopisetty A Ganesh BB Sheng JR Prabhakar BS. GM-CSF-induced, bone-marrow-derived dendritic cells can expand natural Tregs and induce adaptive Tregs by different mechanisms. J Leukoc Biol (2011) 89(2):235–49. 10.1189/jlb.0310154 21048215 PMC3024903

[65] Loubaki L Chabot D Bazin R. Involvement of the TNF-alpha/TGF-beta/IDO axis in IVIg-induced immune tolerance. Cytokine (2015) 71(2):181–7. 10.1016/j.cyto.2014.10.016 25461397

[66] Maldonado RA von Andrian UH. How tolerogenic dendritic cells induce regulatory T cells. Adv Immunol (2010) 108:111–65. 10.1016/B978-0-12-380995-7.00004-5 21056730 PMC3050492

[67] Kuipers H Lambrecht BN. The interplay of dendritic cells, Th2 cells and regulatory T cells in asthma. Curr Opin Immunol (2004) 16(6):702–8. 10.1016/j.coi.2004.09.010 15511661

[68] Kaufman GN Massoud AH Audusseau S Banville-Langelier AA Wang Y Guay J Intravenous immunoglobulin attenuates airway hyperresponsiveness in a murine model of allergic asthma. Clin Exp Allergy (2011) 41(5):718–28. 10.1111/j.1365-2222.2010.03663.x 21255135

[69] Yamamoto M Kobayashi K Ishikawa Y Nakata K Funada Y Kotani Y The inhibitory effects of intravenous administration of rabbit immunoglobulin G on airway inflammation are dependent upon Fcgamma receptor IIb on CD11c(+) dendritic cells in a murine model. Clin Exp Immunol (2010) 162(2):315–24. 10.1111/j.1365-2249.2010.04243.x 20819092 PMC2996599

[70] Qian J Zhu J Wang M Wu S Chen T. Suppressive effects of intravenous immunoglobulin (IVIG) on human umbilical cord blood immune cells. Pediatr Allergy Immunol (2011) 22(2):211–20. 10.1111/j.1399-3038.2010.01049.x 20880351

[71] Bayry J Lacroix-Desmazes S Carbonneil C Misra N Donkova V Pashov A Inhibition of maturation and function of dendritic cells by intravenous immunoglobulin. Blood (2003) 101(2):758–65. 10.1182/blood-2002-05-1447 12393386

[72] Samuelsson A Towers TL Ravetch JV. Anti-inflammatory activity of IVIG mediated through the inhibitory Fc receptor. Science (2001) 291(5503):484–6. 10.1126/science.291.5503.484 11161202

[73] Trepanier P Aubin E Bazin R. IVIg-mediated inhibition of antigen presentation: predominant role of naturally occurring cationic IgG. Clin Immunol (2012) 142(3):383–9. 10.1016/j.clim.2011.12.014 22281428

[74] Aubin E Lemieux R Bazin R. Indirect inhibition of in vivo and in vitro T-cell responses by intravenous immunoglobulins due to impaired antigen presentation. Blood (2010) 115(9):1727–34. 10.1182/blood-2009-06-225417 19965673

[75] Ohkuma K Sasaki T Kamei S Okuda S Nakano H Hamamoto T Modulation of dendritic cell development by immunoglobulin G in control subjects and multiple sclerosis patients. Clin Exp Immunol (2007) 150(3):397–406. 10.1111/j.1365-2249.2007.03496.x 17900307 PMC2219369

[76] Veldhoen M Hocking RJ Atkins CJ Locksley RM Stockinger B. TGFbeta in the context of an inflammatory cytokine milieu supports de novo differentiation of IL-17-producing T cells. Immunity (2006) 24(2):179–89. 10.1016/j.immuni.2006.01.001 16473830

[77] Pasare C Medzhitov R. Toll pathway-dependent blockade of CD4+CD25+ T cell-mediated suppression by dendritic cells. Science (2003) 299(5609):1033–6. 10.1126/science.1078231 12532024

[78] Andersson JP Andersson UG. Human intravenous immunoglobulin modulates monokine production in vitro. Immunology (1990) 71(3):372–6. 2269476 PMC1384435

[79] Darville T Tabor D Simpson K Jacobs RF. Intravenous immunoglobulin modulates human mononuclear phagocyte tumor necrosis factor-alpha production in vitro. Pediatr Res (1994) 35(4 Pt 1):397–403. 10.1203/00006450-199404000-00004 8047375

[80] Clynes R. IVIG therapy: interfering with interferon-gamma. Immunity (2007) 26(1):4–6. 10.1016/j.immuni.2007.01.006 17241954

[81] Bayry J Bansal K Kazatchkine MD Kaveri SV DC-SIGN and alpha2,6-sialylated IgG Fc interaction is dispensable for the anti-inflammatory activity of IVIg on human dendritic cells. Proc Natl Acad Sci U S A (2009) 106(9):E24. author reply E5. 19237553 10.1073/pnas.0900016106 PMC2651302

[82] Kaneko Y Nimmerjahn F Madaio MP Ravetch JV. Pathology and protection in nephrotoxic nephritis is determined by selective engagement of specific Fc receptors. J Exp Med (2006) 203(3):789–97. 10.1084/jem.20051900 16520389 PMC2118246

[83] Siragam V Crow AR Brinc D Song S Freedman J Lazarus AH. Intravenous immunoglobulin ameliorates ITP via activating Fc gamma receptors on dendritic cells. Nat Med (2006) 12(6):688–92. 10.1038/nm1416 16715090

[84] De Groot AS Moise L McMurry JA Wambre E Van Overtvelt L Moingeon P Activation of natural regulatory T cells by IgG Fc-derived peptide “Tregitopes”. Blood (2008) 112(8):3303–11. 10.1182/blood-2008-02-138073 18660382 PMC2569179

[85] Cousens LP Najafian N Mingozzi F Elyaman W Mazer B Moise L In vitro and in vivo studies of IgG-derived Treg epitopes (Tregitopes): a promising new tool for tolerance induction and treatment of autoimmunity. J Clin Immunol (2013) 33(Suppl 1):S43–9. 10.1007/s10875-012-9762-4 22941509 PMC3538121

[86] Elyaman W Khoury SJ Scott DW De Groot AS. Potential application of tregitopes as immunomodulating agents in multiple sclerosis. Neurol Res Int (2011) 2011:256460. 10.1155/2011/256460 21941651 PMC3175387

[87] Kaneko Y Nimmerjahn F Ravetch JV. Anti-inflammatory activity of immunoglobulin G resulting from Fc sialylation. Science (2006) 313(5787):670–3. 10.1126/science.1129594 16888140

[88] Anthony RM Wermeling F Karlsson MC Ravetch JV. Identification of a receptor required for the anti-inflammatory activity of IVIG. Proc Natl Acad Sci USA (2008) 105(50):19571–8. 10.1073/pnas.0810163105 19036920 PMC2604916

[89] Chen XX Chen YQ Ye S. Measuring decreased serum IgG sialylation: a novel clinical biomarker of lupus. Lupus (2015) 24(9):948–54. 10.1177/0961203315570686 25672371

[90] Sondermann P Pincetic A Maamary J Lammens K Ravetch JV. General mechanism for modulating immunoglobulin effector function. Proc Natl Acad Sci USA (2013) 110(24):9868–72. 10.1073/pnas.1307864110 23697368 PMC3683708

[91] Washburn N Schwab I Ortiz D Bhatnagar N Lansing JC Medeiros A Controlled tetra-Fc sialylation of IVIg results in a drug candidate with consistent enhanced anti-inflammatory activity. Proc Natl Acad Sci USA (2015) 112(11):E1297–306. 10.1073/pnas.1422481112 25733881 PMC4371931

[92] Anthony RM Nimmerjahn F Ashline DJ Reinhold VN Paulson JC Ravetch JV. Recapitulation of IVIG anti-inflammatory activity with a recombinant IgG Fc. Science (2008) 320(5874):373–6. 10.1126/science.1154315 18420934 PMC2409116

[93] Schwab I Biburger M Kronke G Schett G Nimmerjahn F. IVIg-mediated amelioration of ITP in mice is dependent on sialic acid and SIGNR1. Eur J Immunol (2012) 42(4):826–30. 10.1002/eji.201142260 22278120

[94] Massoud AH Yona M Xue D Chouiali F Alturaihi H Ablona A Dendritic cell immunoreceptor: a novel receptor for intravenous immunoglobulin mediates induction of regulatory T cells. J Allergy Clin Immunol (2014) 133(3):853–63. 10.1016/j.jaci.2013.09.029 24210883

[95] Samsom JN van Berkel LA van Helvoort JM Unger WW Jansen W Thepen T Fc gamma RIIB regulates nasal and oral tolerance: a role for dendritic cells. J Immunol (2005) 174(9):5279–87. 10.4049/jimmunol.174.9.5279 15843524

[96] Guilliams M Bruhns P Saeys Y Hammad H Lambrecht BN. The function of Fcgamma receptors in dendritic cells and macrophages. Nat Rev Immunol (2014) 14(2):94–108. 10.1038/nri3582 24445665

[97] Fiebiger BM Maamary J Pincetic A Ravetch JV. Protection in antibody- and T cell-mediated autoimmune diseases by antiinflammatory IgG Fcs requires type II FcRs. Proc Natl Acad Sci USA (2015) 112(18):E2385–94. 10.1073/pnas.1505292112 25870292 PMC4426441

[98] Othy S Topcu S Saha C Kothapalli P Lacroix-Desmazes S Kasermann F Sialylation may be dispensable for reciprocal modulation of helper T cells by intravenous immunoglobulin. Eur J Immunol (2014) 44(7):2059–63. 10.1002/eji.201444440 24700174

[99] Guhr T Bloem J Derksen NI Wuhrer M Koenderman AH Aalberse RC Enrichment of sialylated IgG by lectin fractionation does not enhance the efficacy of immunoglobulin G in a murine model of immune thrombocytopenia. PLoS One (2011) 6(6):e21246. 10.1371/journal.pone.0021246 21731683 PMC3121734

[100] Leontyev D Katsman Y Ma XZ Miescher S Kasermann F Branch DR. Sialylation-independent mechanism involved in the amelioration of murine immune thrombocytopenia using intravenous gammaglobulin. Transfusion (2012) 52(8):1799–805. 10.1111/j.1537-2995.2011.03517.x 22257295

[101] Campbell IK Miescher S Branch DR Mott PJ Lazarus AH Han D Therapeutic effect of IVIG on inflammatory arthritis in mice is dependent on the Fc portion and independent of sialylation or basophils. J Immunol (2014) 192(11):5031–8. 10.4049/jimmunol.1301611 24760152 PMC4025610

[102] Geijtenbeek TB Gringhuis SI. Signalling through C-type lectin receptors: shaping immune responses. Nat Rev Immunol (2009) 9(7):465–79. 10.1038/nri2569 19521399 PMC7097056

[103] Gringhuis SI den Dunnen J Litjens M van der Vlist M Geijtenbeek TB. Carbohydrate-specific signaling through the DC-SIGN signalosome tailors immunity to *Mycobacterium tuberculosis*, HIV-1 and *Helicobacter pylori*. Nat Immunol (2009) 10(10):1081–8. 10.1038/ni.1778 19718030

[104] Smits HH Engering A van der Kleij D de Jong EC Schipper K van Capel TM Selective probiotic bacteria induce IL-10-producing regulatory T cells in vitro by modulating dendritic cell function through dendritic cell-specific intercellular adhesion molecule 3-grabbing nonintegrin. J Allergy Clin Immunol (2005) 115(6):1260–7. 10.1016/j.jaci.2005.03.036 15940144

[105] Anthony RM Wermeling F Ravetch JV. Novel roles for the IgG Fc glycan. Ann N Y Acad Sci (2012) 1253:170–80. 10.1111/j.1749-6632.2011.06305.x 22288459

[106] Pincetic A Bournazos S DiLillo DJ Maamary J Wang TT Dahan R Type I and type II Fc receptors regulate innate and adaptive immunity. Nat Immunol (2014) 15(8):707–16. 10.1038/ni.2939 25045879 PMC7430760

[107] Crispin M Yu X Bowden TA Crystal structure of sialylated IgG Fc: implications for the mechanism of intravenous immunoglobulin therapy. Proc Natl Acad Sci USA (2013) 110(38):E3544–6. 10.1073/pnas.1310657110 23929778 PMC3780870

[108] Yu X Vasiljevic S Mitchell DA Crispin M Scanlan CN. Dissecting the molecular mechanism of IVIg therapy: the interaction between serum IgG and DC-SIGN is independent of antibody glycoform or Fc domain. J Mol Biol (2013) 425(8):1253–8. 10.1016/j.jmb.2013.02.006 23416198

[109] Kasermann F Boerema DJ Ruegsegger M Hofmann A Wymann S Zuercher AW Analysis and functional consequences of increased Fab-sialylation of intravenous immunoglobulin (IVIG) after lectin fractionation. PLoS One (2012) 7(6):e37243. 10.1371/journal.pone.0037243 22675478 PMC3366990

[110] Yamazaki S Dudziak D Heidkamp GF Fiorese C Bonito AJ Inaba K CD8+ CD205+ splenic dendritic cells are specialized to induce Foxp3+ regulatory T cells. J Immunol (2008) 181(10):6923–33. 10.4049/jimmunol.181.10.6923 18981112 PMC2814590

[111] Tha-In T Metselaar HJ Bushell AR Kwekkeboom J Wood KJ. Intravenous immunoglobulins promote skin allograft acceptance by triggering functional activation of CD4+Foxp3+ T cells. Transplantation (2010) 89(12):1446–55. 10.1097/TP.0b013e3181dd6bf1 20463648

[112] Becker C Kubach J Wijdenes J Knop J Jonuleit H. CD4-mediated functional activation of human CD4+CD25+ regulatory T cells. Eur J Immunol (2007) 37(5):1217–23. 10.1002/eji.200636480 17407195

[113] Pashov A Bellon B Kaveri SV Kazatchkine MD. A shift in encephalitogenic T cell cytokine pattern is associated with suppression of EAE by intravenous immunoglobulins (IVIg). Mult Scler (1997) 3(2):153–6. 10.1177/135245859700300218 9291172

[114] Pashov A Dubey C Kaveri SV Lectard B Huang YM Kazatchkine MD Normal immunoglobulin G protects against experimental allergic encephalomyelitis by inducing transferable T cell unresponsiveness to myelin basic protein. Eur J Immunol (1998) 28(6):1823–31. 10.1002/(SICI)1521-4141(199806)28:06<1823::AID-IMMU1823>3.0.CO;2-F 9645363

[115] Andersson U Bjork L Skansen-Saphir U Andersson J Pooled human IgG modulates cytokine production in lymphocytes and monocytes. Immunol Rev (1994) 139:21–42. 10.1111/j.1600-065X.1994.tb00855.x 7927412

[116] Maddur MS Vani J Hegde P Lacroix-Desmazes S Kaveri SV Bayry J. Inhibition of differentiation, amplification, and function of human TH17 cells by intravenous immunoglobulin. J Allergy Clin Immunol (2011) 127(3):e1–7. 10.1016/j.jaci.2010.12.1102 21281961

[117] Cooper N Heddle NM Haas M Reid ME Lesser ML Fleit HB Intravenous (IV) anti-D and IV immunoglobulin achieve acute platelet increases by different mechanisms: modulation of cytokine and platelet responses to IV anti-D by FcgammaRIIa and FcgammaRIIIa polymorphisms. Br J Haematol (2004) 124(4):511–8. 10.1111/j.1365-2141.2004.04804.x 14984503

[118] Mouzaki A Theodoropoulou M Gianakopoulos I Vlaha V Kyrtsonis MC Maniatis A. Expression patterns of Th1 and Th2 cytokine genes in childhood idiopathic thrombocytopenic purpura (ITP) at presentation and their modulation by intravenous immunoglobulin G (IVIg) treatment: their role in prognosis. Blood (2002) 100(5):1774–9. 12176899

[119] Wei S Kryczek I Zou W. Regulatory T-cell compartmentalization and trafficking. Blood (2006) 108(2):426–31. 10.1182/blood-2006-01-0177 16537800 PMC1895488

[120] Iellem A Mariani M Lang R Recalde H Panina-Bordignon P Sinigaglia F Unique chemotactic response profile and specific expression of chemokine receptors CCR4 and CCR8 by CD4(+)CD25(+) regulatory T cells. J Exp Med (2001) 194(6):847–53. 10.1084/jem.194.6.847 11560999 PMC2195967

[121] Matsushima H Takashima A. Bidirectional homing of Tregs between the skin and lymph nodes. J Clin Invest (2010) 120(3):653–6. 10.1172/JCI42280 20179349 PMC2827968

[122] Tha-In T Metselaar HJ Tilanus HW Groothuismink ZM Kuipers EJ de Man RA Intravenous immunoglobulins suppress T-cell priming by modulating the bidirectional interaction between dendritic cells and natural killer cells. Blood (2007) 110(9):3253–62. 10.1182/blood-2007-03-077057 17673603

